# Supplementary figures and images for: Impact of paternal hepatitis B on pregnancy outcomes in couples undergoing assisted reproductive technology treatment: a systematic review and meta-analysis
Source: PeerJ. 2025 Aug 18;13:e19824. doi: 10.7717/peerj.19824 (PMC12369629; doi:10.7717/peerj.19824)

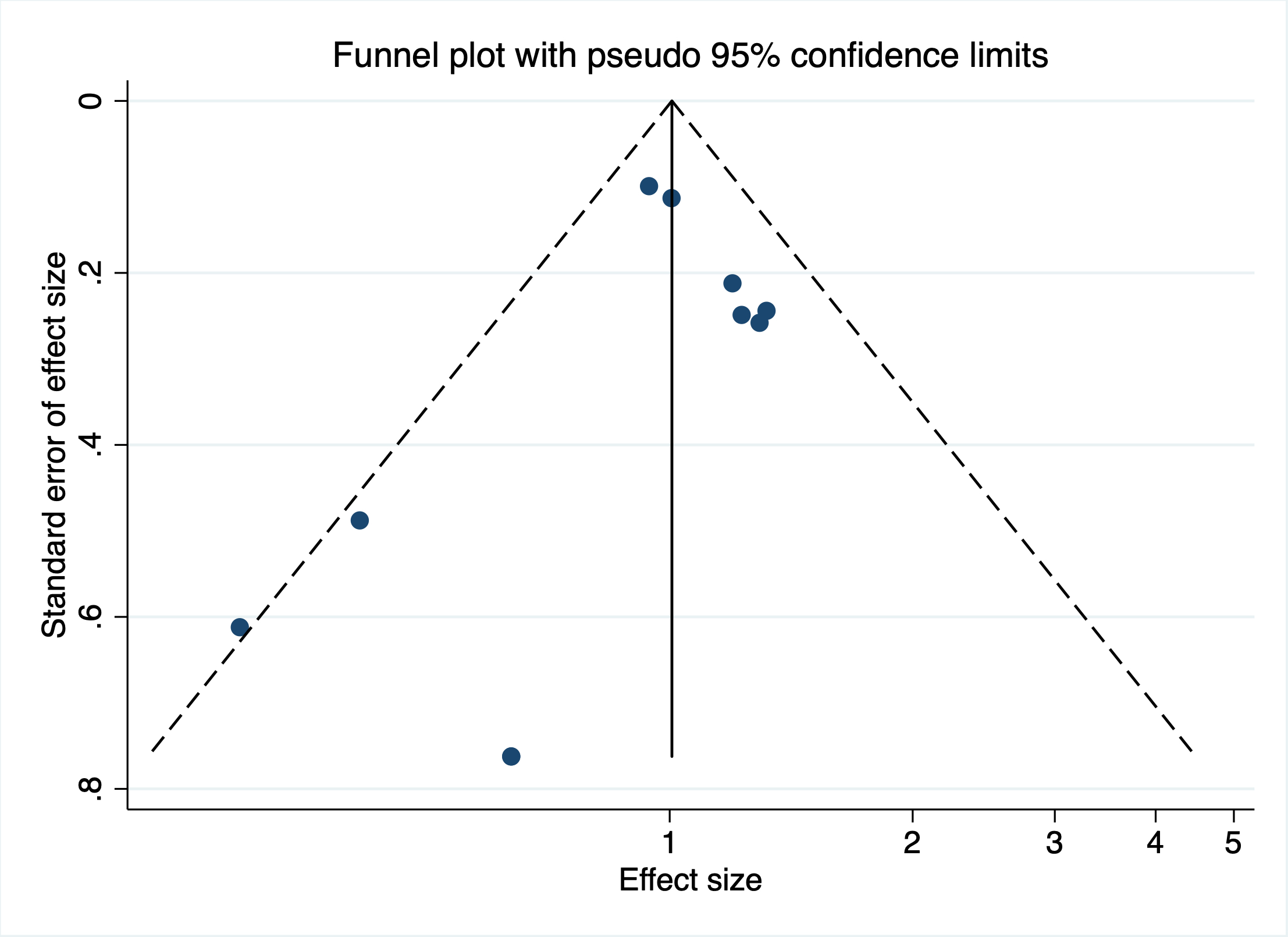

Supplement: Supplemental Information 2 [file peerj-13-19824-s002.jpg]

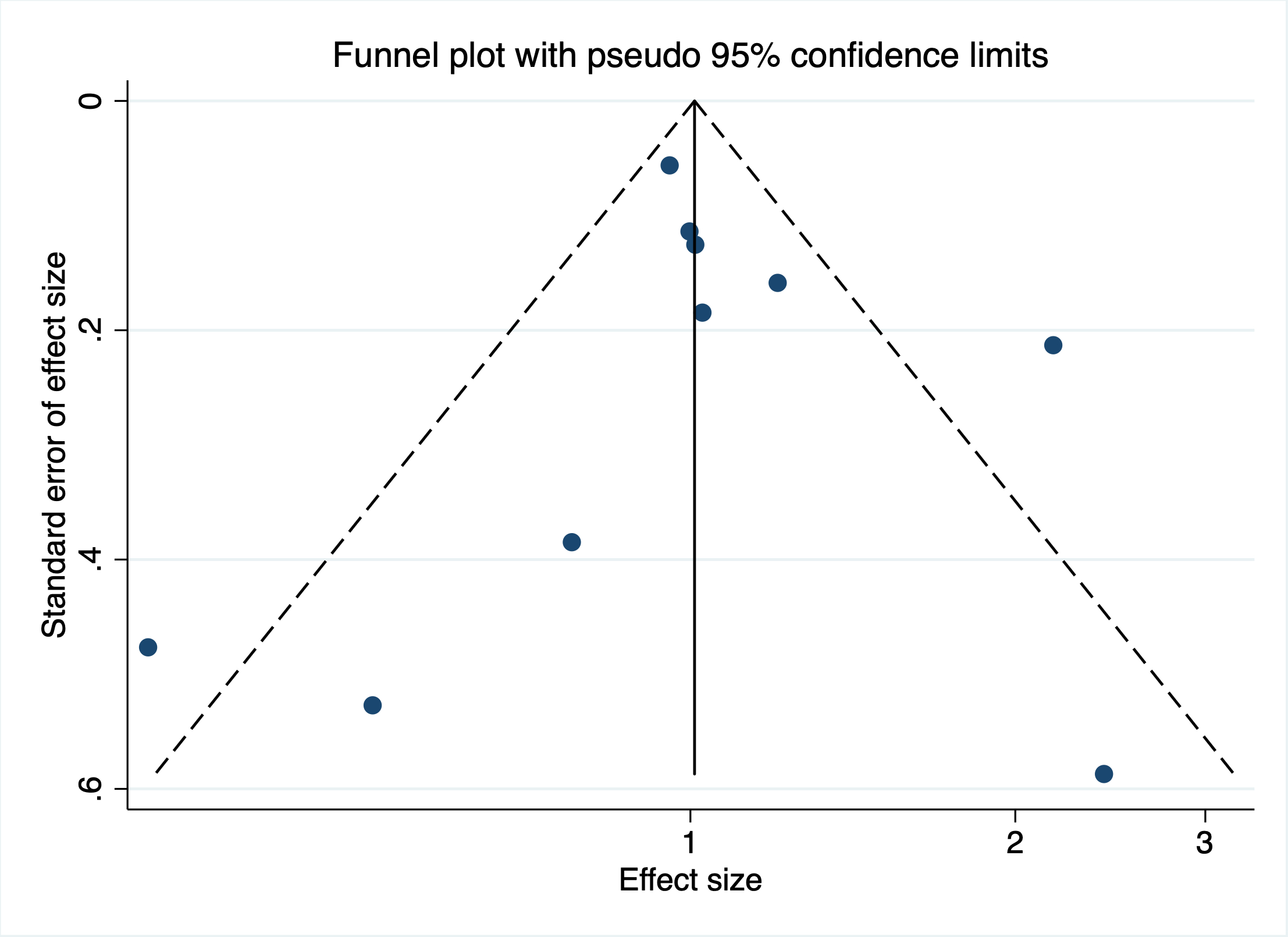

Supplement: Supplemental Information 3 [file peerj-13-19824-s003.jpg]
