# Supplementary material for: Impact of paternal hepatitis B on pregnancy outcomes in couples undergoing assisted reproductive technology treatment: a systematic review and meta-analysis
Source: PeerJ. 2025 Aug 18;13:e19824. doi: 10.7717/peerj.19824 (PMC12369629; doi:10.7717/peerj.19824)
Supplement: Supplemental Information 4 [file peerj-13-19824-s004.docx]

**Supplementary File: Search Strategy**

**PubMed:**

("Hepatitis B"[Mesh]

OR "Hepatitis B virus"[tiab]

OR “viral hepatitis B”[tiab]

OR chronic hepatitis B[tiab]

OR HBV[tiab]

OR HBsAg[tiab]

OR “Hepatitis B surface antigen”[tiab]

OR HBeAg[tiab]

OR “Hepatitis B e antigen”[tiab])

AND

("Reproductive Techniques, Assisted"[Mesh]

OR "Assisted Reproductive Technology"[tiab]

OR ART[tiab]

OR “in vitro fertilization”[tiab]

OR IVF[tiab]

OR “intracytoplasmic sperm injection”[tiab]

OR ICSI[tiab]

OR “intrauterine insemination”[tiab]

OR IUI[tiab])

AND

("Pregnancy Outcome"[Mesh]

OR "pregnancy outcome"[tiab]

OR "pregnancy rate"[tiab]

OR "clinical pregnancy"[tiab]

OR "biochemical pregnancy"[tiab]

OR "ongoing pregnancy"[tiab]

OR "live birth"[tiab]

OR "neonatal outcome"[tiab]

OR "birth weight"[tiab]

OR miscarriage[tiab]

OR "miscarriage rate"[tiab])

Filters: Humans; no language restrictions

**EMBASE**:

('hepatitis b'/exp

OR 'hepatitis b virus':ti,ab,kw

OR 'viral hepatitis b':ti,ab,kw

OR 'chronic hepatitis b':ti,ab,kw

OR hbv:ti,ab,kw

OR hbsag:ti,ab,kw

OR hbeag:ti,ab,kw)

AND

('assisted reproductive technology'/exp

OR 'assisted reproduction'/exp

OR 'in vitro fertilization'/exp

OR 'intracytoplasmic sperm injection'/exp

OR 'intrauterine insemination'/exp

OR art:ti,ab,kw

OR ivf:ti,ab,kw

OR icsi:ti,ab,kw

OR iui:ti,ab,kw)

AND

('pregnancy outcome'/exp

OR 'pregnancy rate':ti,ab,kw

OR 'clinical pregnancy':ti,ab,kw

OR 'biochemical pregnancy':ti,ab,kw

OR 'ongoing pregnancy':ti,ab,kw

OR 'live birth':ti,ab,kw

OR 'neonatal outcome':ti,ab,kw

OR 'birth weight':ti,ab,kw

OR miscarriage:ti,ab,kw

OR 'miscarriage rate':ti,ab,kw)

Limits: human; no language restrictions

**Web of Science:**

TS=(hepatitis B OR HBV OR HBsAg OR HBeAg OR “viral hepatitis B” OR “chronic hepatitis B”)

AND

TS=(“assisted reproductive technology” OR ART OR IVF OR “in vitro fertilization” OR ICSI OR “intracytoplasmic sperm injection” OR IUI OR “intrauterine insemination”)

AND

TS=(“pregnancy outcome” OR “pregnancy rate” OR “clinical pregnancy” OR “biochemical pregnancy” OR “ongoing pregnancy” OR “live birth” OR “neonatal outcome” OR “birth weight” OR miscarriage)

Document Types: Article;

Index: SCI‑EXPANDED, SSCI, A&HCI;

Languages: All;

Species: Humans

**Scopus**:

TITLE‑ABS‑KEY((hepatitis B OR HBV OR HBsAg OR HBeAg OR “viral hepatitis B” OR “chronic hepatitis B”)

AND (“assisted reproductive technology” OR ART OR IVF OR “in vitro fertilization” OR ICSI OR “intracytoplasmic sperm injection” OR IUI OR “intrauterine insemination”)

AND (“pregnancy outcome” OR “pregnancy rate” OR “clinical pregnancy” OR “biochemical pregnancy” OR “ongoing pregnancy” OR “live birth” OR “neonatal outcome” OR “birth weight” OR miscarriage))

Limit to: Articles; Humans; All languages

**CNKI**:

主题=(乙型肝炎病毒 OR HBV OR HBsAg OR HBeAg OR 慢性乙型肝炎)

AND

主题=(辅助生殖技术 OR 体外受精 OR IVF OR ICSI OR 人工授精 OR IUI)

AND

主题=(妊娠结局 OR 生育率 OR 临床妊娠 OR 生化妊娠 OR 持续妊娠 OR 活产 OR 新生儿结局 OR 出生体重 OR 流产 OR 流产率)

来源=中国核心期刊

时限=数据库收录至

**Wangfang data**:

主题=(乙型肝炎病毒 OR HBV OR HBsAg OR HBeAg OR 慢性乙型肝炎)

AND

主题=(辅助生殖技术 OR 体外受精 OR IVF OR ICSI OR 人工授精 OR IUI)

AND

主题=(妊娠结局 OR 生育率 OR 临床妊娠 OR 生化妊娠 OR 持续妊娠 OR 活产 OR 新生儿结局 OR 出生体重 OR 流产 OR 流产率)

来源=所有期刊

时限=数据库收录至
